# Clinical Outcomes and Patients’ Perspectives of Multidisciplinary Psoriasis Management: A Five-Year Retrospective Study

**DOI:** 10.31138/mjr.051223.cop

**Published:** 2024-09-30

**Authors:** Eleni Sotiriou, Katerina Bakirtzi, Ilias Papadimitriou, Eleni Paschou, Dimitra Kiritsi, Nikolaos Kougkas, Eleni Pagkopoulou, Aikaterini Tsentemeidou, Stavros Rizos, Georgia Arvaniti, Themis Chatzi-Sotiriou, Efstratios Vakirlis

**Affiliations:** 1 First Department of Dermatology and Venereology, School of Medicine, Aristotle University of Thessaloniki, Greece; 2 Fourth Department of Internal Medicine, Medical School, Hippokration University Hospital, Aristotle University of Thessaloniki, Thessaloniki, Greece; 3 Psychiatry private practice, External Contractor, Greece; 4 Department of Nutrition, Hippokration University Hospital, Thessaloniki, Greece; 5 Faculty of Arts, Groningen University, Groningen, Netherlands

**Keywords:** psoriasis, psoriatic arthritis, multidisciplinary, patient satisfaction

Dear Editor,

A coordinated, multidisciplinary approach is highly recommended by dermatology societies and desired by patients worldwide to manage psoriasis (Pso) and its comorbidities.^1^ We herein report our 5-year experience in the First Department of Dermatology and Venereology, Aristotle University (Thessaloniki, Greece), after adopting multidisciplinary care in the Psoriasis Department. In this observational, retrospective study, we explored the benefits of a multidisciplinary care model in patients with Pso regarding treatment efficacy, and early detection of comorbidities, particularly psoriatic arthritis (PsA), patients’ quality of life and patient satisfaction.

We recruited all patients attending the Pso combined clinic from June 2018 to June 2023. In 2018, a parallel multidisciplinary care model was developed in the Psoriasis Outpatient Clinic of our dermatology department, incorporating health services between dermatologists, rheumatologists, psychiatrists, and dietitians. In this model, a permanent joint weekly 3-hour visit is scheduled for those patients with Pso who are deemed necessary by the dermatologists to be evaluated by other specialties. All patients referred to the combined clinic were managed jointly by a dermatologist and a rheumatologist, who made appropriate decisions on diagnosis and treatment. Screening for potential PsA in patients with existing Pso was performed through the self-administered five-item Psoriasis Epidemiology Screening Tool (PEST) and/or psoriatic onychia. A PEST value ≥ 3 indicated referral to rheumatologists. Referrals to the combined clinic were also welcomed and internal from either specialty or external from other healthcare units. Once patients were managed in our multidisciplinary unit and their problem was addressed, they returned to the reference specialist for standard follow-up.

Demographics, clinical features, and clinimetrics [Psoriasis Area Severity Index (PASI), Nail Area Psoriasis Severity Index (NAPSI), Dermatology Life Quality Index (DLQI), axial/peripheral involvement, Disease Activity Index for Psoriatic Arthritis (DAPSA) disease activity], history of Pso or PsA, body mass index (BMI), comorbidities, and previous and present treatments including conventional systemic or biologic Pso regimens, conventional synthetic disease-modifying antirheumatic drugs (csDMARDS) and/or biological disease-modifying antirheumatic drugs (bDMARDs) were recorded at baseline and the follow-up visits, as required.

Patients who attended the combined clinic were asked to rate their experience by completing a questionnaire at baseline and regularly at follow-up visits regarding their satisfaction. A 5-point Likert scale was used (1: the worst possible – 5: the best possible). At the end of the questionnaire were also available free-text boxes.

The study was conducted per the Helsinki Declaration and obtained institutional approval. All subjects provided informed consent to participate in the study. Descriptive statistics using mean and standard deviation (SD) were calculated.

Over the 5 years, 721 patients were managed in the Pso combined clinic. Demographics and detailed results are presented in **Table 1**. The utilisation rate of the multidisciplinary unit was 48.0% (721/1503). The mean follow-up visits per patients per year were 4 ±2.8 for the rheumatologic re-evaluation, 6 ±3.1 for the dietitian re-evaluation and 7 ±5.2 for the psychiatric re-assessment.

**Table 1.** Characteristics of patients and clinical outcomes after the multidisciplinary psoriasis disease management between 2018–2023.

**Demographics**	
Age (Mean; ±SD in years)	53 (±17)
Male	41.89% (302/721)
Pso duration (Mean; ±SD in years)	16 (±14)
PsA duration (Mean; ±SD in years)	1 (±2)
BMI (Mean; ±SD)	24.8 (±12.5)

**Timely interventions between 2018–2023**	
Newly diagnosed PsA	79.47% (418/526)
Psychiatric medical intervention	22.22% (8/36)
Weight-loss diet program initiation ≥5% BMI reduction	100% (209/209)
	35.89% (75/209)

**Changes in systemic treatments between 2018–2023**	
Without changes	52.98% (382/721)
Molecule change	47.02% (339/721)
Due to PsA	99.12% (336/339)
Due to Major Depression diagnosis	0.88% (3/339)
Newly initiated biologic DMARDs	72.92% (245/336)
Newly initiated or up-titration of conventional DMARDs	27.08% (91/336)

**Clinimetrics**		
	**Baseline**	**52 weeks after combined management**
≥5% BMI reduction	n/a	35.89% (75/209)
PASI (Mean; ±SD)	9.2 (±2.8)	2.1 (±1.3)
tNAPSI	8.9 (±5.3)	1.4 (±0.8)
DAPSA (Mean; ±SD)	12.8 (±5.0)	3.3 (±6.0)
DLQI (Mean; ±SD)	11 (±7.0)	1 (±3.0)

BMI: Body Mass Index; DAPSA: Disease Activity Index for Psoriatic Arthritis; DMARDs: disease-modifying antirheumatic drugs; DLQI: Dermatology Life Quality Index; PASI: Psoriasis Area Severity Index; PsA: Psoriatic Arthritis; Pso: Psoriasis; SD: Standard Deviation; tNAPSI: target Nail Psoriasis Severity Index

Early detection of PsA in Pso patients was 79.47% (418/526). Treatment modifications were reported for 63.88% (336/526) of Pso-PsA patients: 72.92% (245/336) were newly initiated bDMARDs, while 27.08% (91/336) were initiation or up-titration of csDMARDs. Among patients referred for a psychiatry visit, therapeutic adjustments were needed in 22.22% (8/36). All patients (209/721; 28.99%) referred to dietitians followed a weight-loss program, of which 35.89% (75/209) achieved a clinically meaningful ≥5% BMI reduction.

Most patients (97.92%; 706/721) rated the experience of the combined clinic as excellent-5 **(Figure 1)**. Positive free-text remarks included time efficiency, thorough evaluation, a sense of security, and better disease awareness; negative comments were too many people in the room and lack of privacy. Attempts for combined care units for Pso patients have shown some evidence of success.^2–5^ Regarding PsA, our experience showed that, because of consistent PEST evaluation and psoriatic onychia screening during the follow-up visits by dermatologists, the early detection of such comorbidity was substantially high among patients with psoriasis. This highlights that, even though approximately 30% of PsA patients still experience diagnostic delays of more than 2 years, even in urban areas, such a screening approach would improve the diagnosis time.^6^ Moreover, the evident reduction of DAPSA and PASI scores simultaneously suggests that holistic management of a patient from the diagnosis to timely treatment is beneficial in significant aspects of psoriasis disease, namely the skin and joints.

**Figure 1.**
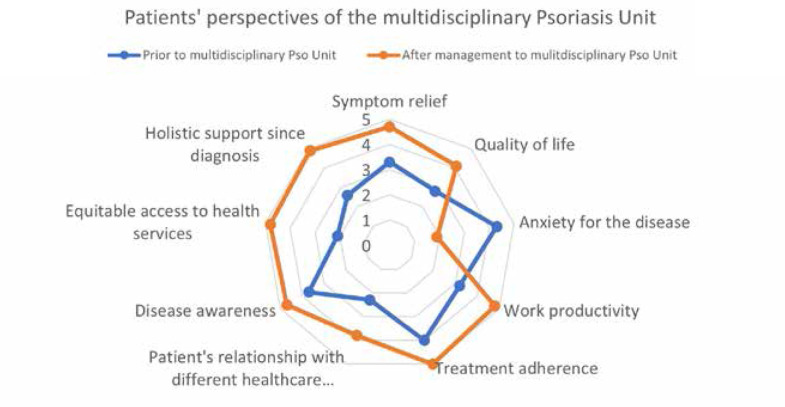
Differences regarding patients’ outlook in certain areas before and after the multidisciplinary approach.

Nonetheless, there has yet to be a proposal on standards of care and quality indicators for the multidisciplinary care of psoriatic disease. The structured cooperation between different specialties could address diagnostic delays and low adherence and improve disease surveillance and outcomes.

## Data Availability

The data that support the findings of this study are available from the corresponding author, K.B., upon reasonable request.
